# Quality by design in public healthcare: a rapid development framework for future pandemics

**DOI:** 10.3389/jpps.2026.16451

**Published:** 2026-06-25

**Authors:** Niranjan Panda, Madhusudan Maity, Vijay Kotra, Bikash Ranjan Jena, Debashish Ghose, Gouri Sankar Jena

**Affiliations:** 1 School of Pharmacy, The Neotia University, Saisha/Kolkata, West Bengal, India; 2 Department of Pharmaceutical Chemistry, Faculty of Pharmacy, Quest International University, Ipoh, Malaysia; 3 Department of Pharmaceutical Sciences, School of Health Science, NIST University, Berhampur, Odisha, India

**Keywords:** AQbD, pandemic, patient-centric, QbD, regulatory framework

## Abstract

The growing number of pandemics and emergencies traversing the world in recent decades has illustrated the vulnerability of the conventional pharmaceutical development process and the necessity of an efficient, robust, and patient-driven pandemic-ready development model. QbD is a scientifically proven proactive approach that has been comprehensively examined recently for its potential to effectively optimize formulations in an accelerated time frame. Risk factor assessment and constant surveillance serve to maintain the product quality & standards before the final stage of large-scale production and offer regulatory pliability. The extension of this method, AQbD, paired side by side also assists in accelerated assessment and quality assurance which is a critical component in any global circumstance. In recent years studies have been done by applying this novel approach, but very little has been reported on the establishment of this approach as a Pandemic-Ready Development Framework. This mini review addresses the regulatory basis alongside the key principles of QbD, which aligns its working approach as optimally compatible with any pandemic situation. Furthermore, it emphasizes the widespread implementation of this approach as AQbD, which stands for swift testing coupled with quality assurance standards. It also focuses on the limitations and critical areas where QbD can be applied to cope with existing demands in near future. Further, it applies this approach in several formulation developments pertinent to pandemic or endemic conditions and discusses the challenges of implementation and future directions. Overall, this discussion stresses the potential of this approach as a very promising framework for future pandemics.

## Introduction

The development of a novel therapeutic molecule is a very complex and time-intensive process due to the various phases, starting from the discovery to the final regulatory approval. But the global health emergencies like the COVID-19 pandemic, the Zika virus epidemic in Brazil, and the Ebola virus epidemic in West Africa highlight the limitations associated with the conventional pharmaceutical development and quality assurance paradigms. The unpredicted need for vaccines, therapeutics, and analytical testing exposed the weakness of the conventional system in emergency preparedness. The pandemic also demands rapid decision-making and large-scale production, but the traditional approach failed to fulfill all the critical requirements. Thus, the global health crisis also signifies the urgent need for a Pandemic-ready drug development framework for efficiently dealing with this condition [1]. Building on this concept, Quality by Design (QbD) and Analytical Quality by Design (AQbD) are systematic, science- and risk-based frameworks developed to enhance quality, consistency, and efficiency in pharmaceutical product and analytical method development. QbD usually focuses on developing quality into the product and the manufacturing process through predefined objectives, process understanding, and risk management. QbD also helps the researcher to effectively determine the critical quality attributes (CQAs) and critical process parameters (CPPs) prior to the final development. Further, this strategy helps to develop the design space (DS) to satisfy the quality requirements of the final product. This strategy helps in a smooth regulatory submission and provides flexibility during the manufacturing and post-approval changes, which are major challenges in the conventional development process [2]. In the case of pandemic-readiness, QbD offers significant advantages. Integration of this approach in both the process and product design helps to reduce the uncertainty associated with the scale-up and the time required for the processing. On the other hand, the risk assessment (RA) tools of QbD help in early detection and handling the hazards during product development, which is critical during pandemic situations [3]. On the other hand, AQbD follows similar principles to analytical methods, which helps in ensuring or ensures robustness and reliability throughout the method’s lifecycle. Analytical methods for testing are also a critical parameter as it utilized largely in the biopharmaceutical as well as vaccine industries. Traditional analytical method development is time-consuming and inconsistent. The AQbD principles help to address the gap in conventional methods by applying a systemic approach aligned with the regulatory requirements [4]. Overall, both the QbD and AQbD are crucial assets in emergency situations where saving time is similar to saving a life. This current review focused on the role of QbD as a pandemic-ready development framework. It provides the overall description of core principles and regulatory foundations of the QbD, role in pandemic preparedness, AQbD for rapid testing and quality assurance, and different formulation platforms for pandemic preparedness, which apply the QbD approach for the development. Further, it highlights the Implementation Challenges, current status, and future direction of this critical approach.

## Core principles and regulatory foundations of QbD

**FIGURE 1:**
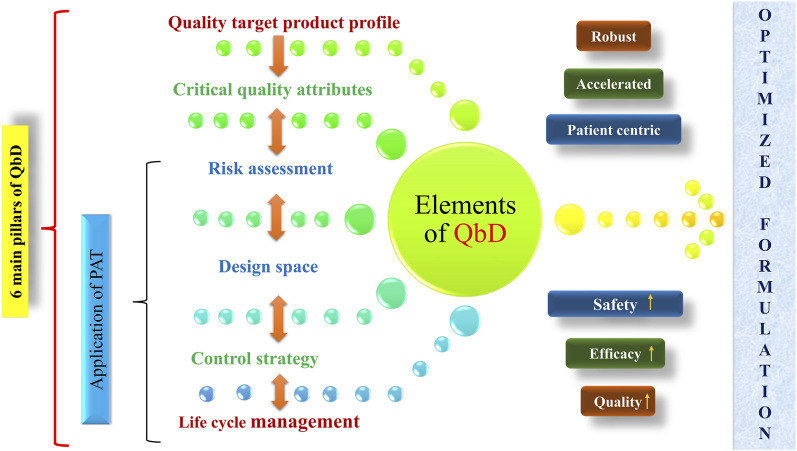
Schematic illustration of key components of the QbD approach.

QbD is usually specified by the International Council for Harmonization (ICH) Q8(R2) guideline as “a systematic approach to development that begins with predefined objectives and emphasizes product and repeated twice process understanding and process control, based on sound science and quality risk management” [5]. This definition signifies the potential of this approach for ensuring the standard of any product from the very beginning, rather than depending upon the final product’s testing. The fundamental principles of QbD include the formation and development of effective control strategies, with a primary focus on continuous improvement throughout the product lifecycle. This core principle is generally grounded in the 6 main pillars of QbD ideology, which can facilitate the development of patient-centric novel optimized formulations, as depicted in Figure 1.

All these 6 key elements are interrelated and focused on the continuous development of quality products, considering the possible risks throughout the product lifecycle [6, 7]. The overall motto of this approach is to make correct, quick, and precise decisions, which makes the method relevant for emergency preparedness.

The ICH has issued several harmonized guidelines as a modern regulatory foundation of this approach. ICH Q8–Q11 guidelines make a major change in thinking in pharmaceutical development (from testing quality at the end to designing quality from the start) [8]. ICH Q8, Pharmaceutical Development, provides the overall concept of DS. ICH Q9, Quality Risk Management, provides the framework for assessing the risk [9]. ICH Q10, Pharmaceutical Quality System and ICH Q11, Development and Manufacture of Drug Substances further strengthened this framework [10, 11]. Progressive expansion of the regulatory framework further includes several additional guidelines, such as ICH Q12, ICH Q13 and ICH Q14. This further supports the integration of advanced manufacturing and modern analytical techniques within the QbD framework. Overall, these harmonized guidelines provide an inclusive shift towards proactive and science-based quality assurance [8].

## QbD as a pandemic-ready development model

This proactive approach supersedes or succeeds the traditional quality by testing models. It supports predictability and regulatory flexibility, which is most essential during emergencies like pandemics, where rapid decision-making, product development, and scale-up is most critical steps. This modern framework provides the details of material attributes (MAs) and process parameters. Further, it correlates how these parameters influence the critical quality attributes (CQAs). Overall, this framework provides a science-backed DS, which supports applications of different modern technological platforms like continuous manufacturing and Process Analytical Technology (PAT). This helps in real-time monitoring and ensures that the CQAs are within the acceptable limits. This approach reduces the chance of batch failure, minimizes variability between batches, and streamlines regulatory acceptance or approval, making it an essential tool for pandemic preparedness [12, 13].

Design of Experiments (DoE) and different RA tools like Failure Mode and Effects Analysis (FMEA) are the methodologies generally used for smooth process understanding. This flexibility is another key advantage of the QbD, which further helps to find out the optimal operational range before performing any kind of full-scale manufacturing. Any changes within this operating range also require minimal regulatory adherence. So, this approach helps in a smooth transition from the development phase to the production phase. This approach of QbD supports the rapid commercialization of any product during the pandemic situation, where minor changes and adjustments in formulation are required [14, 15].

**FIGURE 2:**
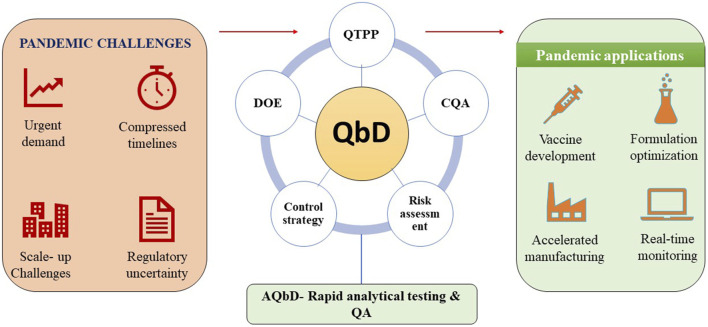
Schematic illustrating QbD and AQbD collectively enable pandemic readiness.

A recent study by van de Berg et al. used QbD based frame work for the rapid development of an RNA vaccine to combat emerging infectious diseases. Their mechanical modeling approach developed a robust design space and achieved RNA yields above 4.34 g/L with improved consistency and minimum variability. This approach helps in rapid process optimization and improves the production volume while maintaining the quality standards of the final product. This signifies the potential of QbD-based approach for rapid optimization and large-scale production of such vaccines during pandemic conditions [16]. The traditional pathways ensure that “quality” is achieved by testing the finished product (Quality by Testing). Quality is engineered into the product and production process throughout development. It’s a “fixed” procedure and any divergence usually demands a new validation cycle. Empirical, we have only a vague grasp of how many factors interact. “High chance of rejected batches if they don’t pass final specs.” The QbD based methodology ensures that quality is designed into the product and its manufacturing process in development. A “flexible” method works inside a specified Design Space identification comprehensive multivariate insight into the effect of process factors on quality. Low risk zone is available. Real-time monitoring allows modifications to avoid batch failures [17]. The schematic illustration about the QbD/AQbD approach has been elucidated in Figure 2.

## AQbD for rapid analytical testing and quality assurance

The pandemic demands the rapid development of vaccines and therapeutics, which highlights the need for a validated analytical method to assess these therapeutics. The rapid development of quality products for pandemic preparedness highlights the potential of AQbD. This enables the implementation of the QbD paradigm in the development of analytical methods, which ensures the robustness, reliability and accuracy of any analytical procedures [17]. The AQbD approach started by defining the Analytical Target Profile (ATP); this provides a brief summary of the desired performance characteristics of an analytical procedure. Depending upon this baseline set by ATP, the Method Operable Design Region (MODR) is identified by applying DOE and RA tools. Within the MODR, the analytical performance meets the ATP criteria. This systematic and science-backed approach improves the method’s robustness, minimizes variability, and helps in rapid analytical development by speeding up this process [18].

A recent review by Rajendra Kotadiya demonstrates the application of the AQbD framework in around 52 research studies from 2014 to 2025. This review further summarizes how AQbD helps to improve the ruggedness of the method, reduce method transfer failure, and ensure accurate analytical testing even under an accelerated time period, which is crucial during emergencies. This AQbD workflow helps in the smooth implementation of the analytical procedure by reducing the risk of out-of-specification results and improving the reliability of the methodology. This further ensures the quality of the product [19].

Another study by Nuli MV et al. reported the implementation of the AQbD approach for chromatographic method optimization. The study demonstrates that this approach not only improves the method performance but also helps to reduce the time required for development and support, establishing a stable RP-UPLC technique for analysis of nivolumab and relatlimab formulations [20].

Regulatory guidelines are also supporting the implementation of AQbD for rapid analytical testing and quality assurance. The ICH Q14 guideline provides a framework for analytical procedure development using risk-based approaches [18]. Along with Q14, other ICH guidelines facilitate smooth regulatory processing between industry and regulatory agencies for the design and optimization of accurate and flexible analytical methods, which are essential during emergency situations where rapid development is desirable. Overall, the AQbD approach improves the reliability and robustness of analytical methods and serves as a potential framework for rapid analytical testing and quality assurance during public health emergencies.

## QbD-driven formulation platforms for pandemic preparedness

Implementation of this proactive approach in different formulation optimization provides predictable performance, smooth scalability, and regulatory flexibility. This science-backed approach helps in the rapid optimization of formulation during the development phase. This is the primary requirement during any pandemic situation. This section specifically discusses the application and efficacy of the QbD-based strategy in various formulations, which are particularly relevant based on their application in different pandemic scenarios. Integration of QbD principles in different formulation developments, such as optimization of lipid nanoparticles, RNA-based vaccines, nanocarriers, and microemulsions (key delivery systems during emergency responses), and their outcomes are summarized in Table 1 [21–27].

**TABLE 1:** Recent Case studies on QbD-Based Formulation Relevant to Pandemic-Ready Platforms.

Delivery system	QbD-based strategy adopted	Key findings	References
1. Lipid nanoparticle for RNA delivery	DOE-based optimization of the nanoparticle system; establishment of design space	This study reported rapid optimization of the formulation and scalable vaccine manufacturing	[21]
2. RNA vaccine manufacturing	Mechanistic bioprocess modeling and probabilistic design space under the QbD framework	Studies demonstrate high-yield, reproducible RNA production with accelerated scale-up capability	[16]
3. Microemulsion	FMEA-based RA, DoE	Stable microemulsion system, with improved drug loading, stability, and sustained drug release	[22]
4. DNA vaccine purification process	Central composite DoE and modeling to characterize CQA and process understanding	Established predictive models for DNA vaccine purification, helpful for large-scale purification and production	[23]
5. Mucoadhesive minitablets of cefuroxime axetil	Determination quality target product profile (QTPP), CQAs, box–Behnken design using ANOVA, DoE optimization	Improve bioavailability and high control over the drug’s release, flexible adjustment of dose and release profile, high drug content, mucoadhesion and swelling profile	[24]
6. mRNA vaccine production	Bayesian optimization technique, model interpretability Techniques	Improve the mRNA production twofold, cost-effectively, and improve the manufacturing capacity	[25]
7. QbD for mRNA platform purification	Application of QbD framework for the development of the DS and process parameters is optimized	Helps to achieve more than 90% mRNA recovery; greater than 99% purity; 15% lower cost compared to the conventional method	[26]
8. Polymeric nanoparticles of cinacalcet hydrochloride	QBD-based optimization, identification of quality target product profiles (QTPPs) and CQAs, BBD response surface design for nanoparticle optimization	Sustained drug release profile, PK parameters increased 2.9 to 3-fold, improved therapeutic efficacy and bioavailability	[27]
9. QbD based RNA vaccine production	Quality by design for enabling RNA platform production processes	Current knowledge on CQAs of RNA-based products, with product immunogenicity and instability being the main risks, as well as the intimate relationships between product and process	[28]
10. QbD based RNA vaccine and therapeutic manufacturing	Quality by digital design for developing platform RNA vaccine and therapeutic manufacturing processes	QbD approach in developing and optimizing unit operations such as *in vitro* transcription, tangential flow filtration, affinity chromatography, and lipid nanoparticle (LNP) formulation in mRNA vaccine manufacturing	[29]

The overall summary of recent reviews and research data from Table 1. Signifies the potential of QbD-based framework and its successful application in the formulation development and highlights the efficacy and reproducibility of this approach, along with minimizing the time required for product development. Also, the modern approaches are using various industry-ready modern statistical software (Design Expert, Fusion Product Development, Auto-chrome MDS, Minitab) for analytical research and development using the AQbD and multivariate analysis. The following software of QbD utilizing CADD principles can be strongly implemented in pharmaceutical and biotech research and development labs (R&D) for the rapid production of vaccines and other pandemic-ready therapeutics [30].

## Limitations of QbD adoption, regulatory bottlenecks, cost implications and challenges

QbD is primarily concerned with the creation of the design space via regular experimentation (e.g., Design of Experiments, or DoE). The biggest drawback of QbD adoption is the need for full knowledge of Critical Material Attributes (CMAs) and Critical Process Parameters (CPPs), which is not time-constrained. Data creation connected to clinical trials during pandemic situations is easier to approach than the real production time scale. Therefore, the time required for design space mapping might delay the development of life-saving therapies. In an active pandemic, the susceptibility of the target results in fast turnover and the emergence of variants that might alter the needed effectiveness criteria of dosage forms. Consequently, if the QTPP’s design space changes significantly, it will not be correctly identified, and a “restart” would be necessary. Regulatory organizations (FDA, EMA, etc.) are supportive of QbD, although the legal frameworks associated with Emergency Use Authorizations (EUA) are generally centered on conventional “Fixed Process” validation, not “Flexible Design Space” validation [31]. Companies may not get clear instructions on how much QbD data is “enough” for an emergency submission and return to older, less efficient validation procedures to ensure a speedier regulatory route. While QbD requires more capital expenditure at the onset of a pandemic, it works as a financial insurance policy against the catastrophic costs of manufacturing failures and regulatory delays. The implementation of QbD relies on new skill sets in multivariate data analysis, statistical modeling and risk management, which some organizations now consider out of reach. Many pharmaceutical firms do not possess the requisite equipment and data-management systems for real-time monitoring, knowledge management and Process Analytical Technology (PAT) for a complete QbD implementation. Companies sometimes believe authorities (such as FDA) don’t provide enough clear direction on how to realistically apply QbD, especially about acceptable criteria for analytical method replacement [32].

## Conclusion and future directions

The QbD-based approach shifts the development framework from traditional to a proactive and knowledge-based approach based on assessing risk, establishing DS, and managing the life cycle. This mini-review highlights the potential of this framework during any endemic and pandemic situations, where accelerated production, scale-up, and regulatory flexibilities are the key factors to consider within a very short time period. Besides QbD, the AQbD-based approach also plays a very crucial role during the pandemic by enabling rapid analytical testing and quality assurance. Furthermore, the application of various QbD tools is also used in the optimization and development of pandemic-ready therapeutics. Moreover, the modern regulatory guidelines also backed this approach and made this process smoother and more effective. Despite these advantages, this approach faces several challenges, such as a lack of harmonization in regulatory requirements across the world, the QbD approach not being widely adopted for academic research, and variability in the application of its principles. To standardize this framework for implementation in a pandemic situation, future research needs to focus on collaboration among academics, industry, and regulatory bodies. Further, the future application of the QbD-based approach in different nanoformulations and vaccine development will also help in the more effective and rapid formulation development during emergency conditions and will help to establish this approach as a Pandemic-ready development framework.
